# Evaluation of Phytochemical Contents and In Vitro Antioxidant, Anti-Inflammatory, and Anticancer Activities of Black Rice Leaf (*Oryza sativa* L.) Extract and Its Fractions

**DOI:** 10.3390/foods10122987

**Published:** 2021-12-03

**Authors:** Chorpaka Thepthanee, Chan-Chiung Liu, Hsu-Sheng Yu, Ho-Shin Huang, Chia-Hung Yen, Yen-Hsien Li, Maw-Rong Lee, Ean-Tun Liaw

**Affiliations:** 1 Department of Food Science, National Pingtung University of Science and Technology, Pingtung 91201, Taiwan; chorpaka.thep@gmail.com (C.T.); ccliu@mail.npust.edu.tw (C.-C.L.); hsyu@mail.npust.edu.tw (H.-S.Y.); 2 R&D Center, King Herb BioMed, Tainan 71201, Taiwan; adinol.huang@gmail.com; 3 Department of Biological Science and Technology, National Pingtung University of Science and Technology, Pingtung 91201, Taiwan; chyen0326@mail.npust.edu.tw; 4 Department of Chemistry, National Chung Hsing University, Taichung 420, Taiwan; liwenshin@dragon.nchu.edu.tw (Y.-H.L.); mrlee@dragon.nchu.edu.tw (M.-R.L.); 5 Instrument Center, Office of Research and Development, National Chung Hsing University, Taichung 420, Taiwan

**Keywords:** black rice leaves, antioxidant, anti-inflammatory, phenolic composition, apoptosis

## Abstract

Black rice leaves (*Oryza sativa* L.) are a major part of rice straw left in open fields after rice harvest as agricultural waste. In this study, crude ethanolic extract (CEE) and various solvent fractions (hexane (Hex), ethyl acetate (EtOAc), *n*-butanol (*n*-BuOH), and aqueous fractions) of black rice leaves were investigated for their bioactive compound contents as well as antioxidant, anti-inflammatory, and anticancer activities. The results demonstrated that among all the fractions, the *n*-BuOH fraction presented the greatest contents of total phenolics and flavonoids, while anthocyanins were found to be abundant in the *n*-BuOH and aqueous fractions, which also exhibited powerful antioxidant abilities according to DPPH and ABTS radical-scavenging assays and a reducing power assay. Regarding anti-inflammatory activity, CEE and EtOAc reduced the production of NO and cytokine secretion (PGE_2_, IL-6, and IL-1β) but displayed less effect on tumor necrosis factor α (TNF-α) release in lipopolysaccharide (LPS)-induced RAW 264.7 cells. They also significantly decreased iNOS and COX-2 protein expression. Additionally, the phenolics-rich ethyl acetate fraction showed the greatest activity against HepG2 liver carcinoma cells, inhibited cell growth, increased the Sub-G1 population, and induced apoptosis via mitochondrion-dependent mechanisms. In conclusion, black rice leaves, a byproduct of rice, exhibited strong antioxidant, anti-inflammatory, and anticancer capacities and might be useful for application in functional foods and the pharmaceutical industry.

## 1. Introduction

Free radicals are generated in our bodies due to oxidation [1]. An excess of them, especially reactive oxygen species (ROS), leads to oxidative stress that damages not only cellular components such as DNA, lipids, and proteins but also tissues and eventually organs. This plays a crucial role in the progression of chronic and degenerative diseases such as diabetes, cardiovascular disease, Alzheimer’s disease, inflammation, and cancers [1,2]. Antioxidants are compounds that inhibit oxidation by donating electrons to free radicals, thereby stabilizing them. Thus, they can help to minimize the numbers of free radicals [2].

Inflammation is a simple biological process but a complex immune response [3]. It plays a vital role in combating foreign bodies or things that injure cells or tissues and can be divided into chronic and acute inflammation [3]. Numerous studies provide evidence that chronic inflammation is associated with multiple phases of carcinogenesis, such as cellular transformation, apoptosis, survival, proliferation, invasion, angiogenesis, and metastasis [4]. In response to inflammatory stimuli, macrophages emit a range of inflammatory mediators such as nitric oxide (NO) and prostaglandin (PGs), as well as pro-inflammatory molecules such as interleukin-6 (IL-6), interleukin-1β (IL-1β), and tumor necrosis factor (TNF-α) [5]. Previous research found that several natural antioxidant compounds such as phenolic groups serve an essential function in the elimination of ROS, as well as being able to control the secretion of numerous pro-inflammatory mediators during chronic inflammation [6]. Phenolic compounds are bioactive substances widely found in edible plants that consist of a major group of phenolic acids and flavonoids, which are known to exert several biological functions in antioxidant, anti-inflammatory, and anticancer capacities [7,8,9].

Rice straw is a byproduct of rice production left after the rice grain has been collected by paddy harvesting, and it contains the main parts of the stems, leaves, and spikelet. The yield of rice straw, about 650–975 million tons annually around the world [10], varies widely among rice cultivars and environmental conditions. Three types are commonly used in agriculture, especially in feed for ruminants and in mushroom and biochar production [11]. Previous reports demonstrated that rice straw shows antioxidant properties, including DPPH and nitric oxide free radical-scavenging activities [12]. These biological activities might be attributed mainly to their bioactive components, including the phenolic acids gallic acid, pyrogallol, caffeic acid, and flavonoids—kaempferol, apigenin, and rutin—as determined by HPLC in three varieties of rice straw [12]. The predominant soluble phenolic, ferulic, and *p*-coumaric acids have also been detected in rice straw cv. Sakha 104 [10]. Interestingly, in some pigmented rice cultivars, their leaves contain purple or black colors. The nutrition values, phytochemical components, antioxidant, biological activities of pigmented and non-pigmented rice grass have been investigated. Pigmented rice leaf juices collected at the jointing stage that contained high anthocyanins content demonstrated greater antioxidant capacity than the green leaf [13]. Moreover, the black rice leaf from Kum Doisaket cultivar possessed the highest DNA protective properties in a concentration-dependent manner [13]. Black glutinous rice grass decreased human T-lymphocyte (Jurkat) cell growth by triggering cell death and caspase 3/7 activity, as well as reducing ROS generation [14].

This study is the first to illustrate the bioactive components in black rice leaves, which are agri-food residues, and their biological activities. The objectives of this study were to determine the in vitro antioxidant, anti-inflammatory, and anticancer capacities of crude extract and its fractions. The bioactive components, phenolic acids, and flavonoids from black rice leaves were also investigated using UV-LC-MS/MS.

## 2. Materials and Methods

### 2.1. Preparation of Plant Materials

Black rice leaves were gathered from the Hualien District Agricultural Research and Extension Station Council of Agriculture (Hualien, Taiwan), In 2019, the black rice leaves were grown and harvested in Hualien, Taiwan. In order to remove contaminants, fresh rice leaves (3 kg) were washed. The leaves were subsequently dried with hot air (40 °C, 24 h) to achieve a moisture content of 7%. After drying in hot air, the sample was powdered, passed through a 100-mesh sieve, and deposited in dry, dark conditions at 4 °C before further use.

### 2.2. Extraction and Fractionation of Bioactive Substances

Phytochemical substances were extracted from black rice leaves after fractionation with different solvent ranging from low to high polarity. Dried powder of the sample (200 g) was extracted thrice with 75% ethanol for 24 h. The extract was then collected on filter paper (Whatman No. 1) under suction and dried at 40 °C, followed by lyophilization for 72 h. The crude ethanolic extract (CEE), with 20.15% yield (*w/w*, dry basis), was soaked in distilled water and then sequentially fractionated in a separatory funnel with hexane, ethyl acetate, and *n*-butanol three times. The resulting solvent fractions were each filtered and concentrated using a rotary evaporator. Four fractions of hexane, ethyl acetate, *n*-butanol, and aqueous fractions were obtained with yields of 12.69%, 3.14%, 28.97%, and 50.29% (*w/w*, dry basis), respectively.

### 2.3. Determination of Total Phenolics, Flavonoids, and Anthocyanins

#### 2.3.1. Total Phenolic Content (TPC)

The TPC of the CEE and its fractions were measured following Folin and Ciocalteu’s methods [15]. In short, 100 μL of standard gallic acid (20–100 μg/mL) or the extracts were combined with 2 mL of 2% sodium carbonate. Folin–Ciocalteu reagent was applied to each test tube after 5 min of incubation, and the tubes were kept without light at ambient temperature for 30 min. The absorption of the solution at 750 nm was measured using an automated microplate reader (SPECTROstar Nano, Ortenberg, Germany). Phenolic content is expressed as milligrams per gram of dried weight (mg GAE/g DW) of gallic acid equivalents.

#### 2.3.2. Total Flavonoid Content (TFC)

The flavonoid contents of the CEE and its fractions were measured using Baker et al.’s [16] method with slight modifications. In short, 250 µL of quercetin standards (40–500 µg/mL) or the extracts were combined with 1.25 mL of distilled water and 75 µL of sodium nitrite (5%), respectively, for 6 min. Then, in a test tube, the solution was incubated without light for 5 min with 150 μL of 10% AlCl_3_. After that, 500 µL of 1 M NaOH was added, followed by 275 µL of distilled water. The absorption of the solution at 510 nm was measured using an automated microplate reader (SPECTROstar Nano, Ortenberg, Germany). The TFC is reported as milligrams of quercetin equivalents per gram of dried weight (mg QE/g DW).

#### 2.3.3. Total Anthocyanin Content (TAC)

The pH-differential method was used to measure the anthocyanin contents of the CEE and its fractions [15]. In test tubes, 280 μL of 0.025 M potassium chloride buffer (pH 1.0) and 280 μL of 0.4 M sodium acetate buffer (pH 4.5) were combined with each extract solution (20 µL), and the mixtures were stored for 10 min in darkness. The absorption at 510 and 700 nm of the mixture was then measured using an automated microplate reader (SPECTROstar Nano, Ortenberg, Germany). The wavelength value (*A*) was determined as follows:

*A* = (*A*_510 nm_ − *A*_700 nm_)_pH 1.0_ − (*A*_510 nm_ − *A*_700 nm_)_pH 4.5_

The TAC is expressed as milligrams of cyanidin 3-glucoside equivalents per one hundred grams of dried weight (mg C3G/100g DW), and was determined according to the following formula:

Anthocyanins content (mg C3G /L) *= A* × 449.2 × *DF* × 10^3^/ (*µ* × *L*)

where 449.2 is the molecular weight of C3G (g/mol), *DF* is the dilution factor, *µ* is the molar extinction coefficient of C3G (26,900 (L cm^−1^ mol^−1^)), *L* is the length of the path in centimeters, and 10^3^ is the factor for conversion from grams to milligrams.

### 2.4. Antioxidant Activity

#### 2.4.1. DPPH Radical-Scavenging Activity

In brief, 200 µL CEE and its fractions solutions were combined with 50 µL of 0.1 mM methanol solution of DPPH; the mixture was incubated without light for 30 min at room temperature, and the wavelength at 517 nm was recorded immediately using an automated microplate reader (SPECTROstar Nano, Ortenberg, Germany) [17]. The data are represented as the extract concentration that inhibited DPPH radicals by 50%. Butylated hydroxyanisole (BHA) was used as a comparative standard.

#### 2.4.2. ABTS^+^ Radical-Scavenging Activity

Briefly, 7 mM ABTS solution was combined with 2.54 mM potassium persulphate and stored without light at 4 °C for 12–16 h to obtain an ABTS^•+^ stock solution. Prior to analysis, the ABTS^•+^ solution mixture was made by adding distilled water to adjust the wavelength at 734 nm to 0.70 ± 0.02. ABTS reagent (180 µL) was added to the sample extract (20 µL). After that, the mixture was incubated without light for 6 min at room temperature, and the wavelength at 734 nm was recorded immediately using an automated microplate reader (SPECTROstar Nano, Ortenberg, Germany) [17]. The results were calculated as the extract concentration that inhibited ABTS radicals by 50%. As a comparative standard, 6-hydroxy-2,5,7,8-tetramethylchroman-2-carboxylic acid (Trolox) was used for comparison.

#### 2.4.3. Reducing Power Assay

Firstly, 500 μL of phosphate buffer (0.2M, pH 6.6) and 500 μL of potassium ferricyanide solution (1%) were combined with each extract solution (500 µL), and incubated for 20 min at 50 °C. After cooling promptly on ice, an equal volume of 10% TCA (trichloroacetic acid) was added and the mixture was centrifuged at 2500 g for 10 min. The supernatant (100 μL) was then loaded to a 96-well plate and each well was filled with distilled water (100 µL) and 0.1% ferric chloride solution (20 µL). The wavelength at 700 nm of the resulting solution was recorded [18]. The results were reported as the effective concentration at which the absorbance is 0.5 for reducing power.

### 2.5. Cell Culture and Sample Treatment

All cell lines were purchased from the BCRC, Bioresource Collection and Research Center (Hsinchu, Taiwan). The RAW 264.7 macrophage cell line and the human hepatoma cancer cell line HepG2 were maintained in high-glucose Dulbecco’s modified Eagle’s medium (DMEM). The normal mouse liver cell line FL83B was cultured in Nutrient Mixture F-12 Ham medium. The cells, cultured in medium with 10% fetal bovine serum (FBS), were maintained in a humidified incubator under 5% carbon dioxide (37 °C).

### 2.6. Cytotoxicity

The samples were resuspended in DMEM and then filtered using a 0.20 µm syringe filter (Pall corporation, Fajardo, Pueto Rico). Cells were plated in 96-well plates (1.0 × 10^4^ cells/well) with 100 μL of DMEM and 10% FBS overnight. The medium was then discarded. RAW 264.7 cells were pre-treated with the CEE and fractions (0–200 µg/mL) for 4 h, followed by LPS activation (100 ng/mL) for 20 h. However, HepG2 and FL83B cells were treated with the CEE and fractions (0–1000 µg/mL) or 5-fluorouracil (25 µg/mL) for 24 h. Twenty microliters of MTT solution (5 mg/mL) was loaded into each well, and the plates were then incubated at 37 °C for 4 h. After the aspiration of the medium and MTT, 100 μL of dimethyl sulfoxide was added and placed on a microplate shaker without light at 100 rpm for 30 min. The wavelength was measured at 570 nm using an ELISA reader.

### 2.7. Determination of NO Production

RAW 264.7 cells were seeded in 24-well plates (1 × 10^5^ cells per well) supplemented with DMEM and 10% FBS, and then maintained for 24 h. They were then pre-treated with the CEE and fractions (0–200 µg/mL) for 4 h, followed by LPS activation (100 ng/mL) for 20 h. Then, 100 µL of the cell culture supernatants were transferred to a 96-well plate and Griess reagent (100 µL) was added into each well, and the plates were then incubated for 15 min. The wavelength was measured at 540 nm using an automated microplate reader, and the nitric oxide concentration of the sample was determined using sodium nitrite (NaNO_2_) as a comparative standard.

### 2.8. Measurement of PGE_2_ and Cytokine (IL-6, IL-1β, and TNF-α) Secretion

RAW 264.7 cells were seeded in 24-well plates (1 × 10^5^ cells per well) for 24 h and pre-treated with or without the CEE and its fractions for 4 h, followed by stimulation with 100 ng/mL LPS for 20 h in order to quantify the amounts of PGE_2_, IL-6, IL-1β, and TNF-α according to the instructions for the ELISA kits. The ELISA kits for IL-6 and TNF-α quantification were obtained from QIAGEN Science (Frederick, MD, USA). The ELISA kits for IL-1β and PGE_2_ quantification were obtained from R&D Systems, Inc. (Minneapolis, MN, USA). The supernatant of the culture medium was collected after centrifugation.

### 2.9. Flow Cytometric Analysis of Cell-Cycle Arrest and Apoptosis

To evaluate the cell-cycle arrest, cells were stained with propidium iodide (PI) using the Propidium Iodide Cytometry Kit (Abcam, Cat. No, ab139418). HepG2 cells were seeded in 6-well culture plates (3 × 10^5^ cells/well) and treated with EtOAc (250, 500, 750, and 1000 µg/mL) or 5-fluorouracil (25 µg/mL) for 48 h. Next, the cells were washed with PBS, harvested using trypsin reagent, and rinsed with phosphate-buffered saline (PBS). After centrifugation, the cells were fixed with ice-cold 70% ethyl alcohol and kept at −20 °C for at least 2 h. The cells were rinsed with PBS and labeled with PI staining solution (200 µL) for 30 min at 37 °C without light. Finally, a flow cytometer was used to measure the DNA content, and analysis was performed with the FlowJo Software (FLOWJO, Ashland, OR, USA).

The FITC Annexin V Apoptosis Detection Kit with PI (Biolegend, Fell, Germany) was used to determine apoptotic cells. The cells were collected in a 15 mL tube and rinsed with cold buffer of cell staining. The suspended cells were labeled with FITC Annexin V accompanied by PI for 15 min without light. Subsequently, the mixture was immediately subjected to flow cytometry analyzer. The data were analyzed using the FlowJo Software (FLOWJO, Ashland, OR, USA) and are reported as percentage of apoptosis.

### 2.10. Western Blot Analysis

RAW 264.7 and HepG2 cells were seeded in a 10 cm^2^ cell-culture dish (1 × 10^6^ cells/well) and treated with or without the CEE and EtOAc for 24 and 48 h. Then, the cells were rinsed thrice with ice-cold PBS and lysed with radioimmunoprecipitation assay buffer containing a protease inhibitor cocktail for 20 min on ice. The mixtures were centrifuged (12,000× *g*) at 4 °C for 10 min. The total protein levels were measured using the Coomassie Plus (Bradford) Assay Kit (Thermo Science, Rockford, IL, USA). Equal volumes of the cell lysates were loaded into 10% sodium dodecyl sulfate polyacrylamide gels for electrophoresis, and then, the proteins were transferred to PVDF membranes by electroblotting (Bio-Rad, Hercules, CA, USA). The membranes were placed in the box, which contained 5% non-fat milk in Tris Buffered Saline with Tween 20 (TBST) and shaken on a microplate shaker at 55 rpm for 1 h. After overnight (4 °C) incubation with the primary antibody, the membranes were soaked with TBST for 10 min three times and soaked with a blot-grade labeled secondary antibody for 1 h at room temperature. Following further rinsing in TBST, the bands of the target protein were visualized using the ECL detection system. All Western blot antibodies were purchased from Cell Signaling Technology, Inc. (Danvers, MA, USA).

### 2.11. Identification of Phenolic Compounds

Catechin as an internal standard (1 mL, 40 µg/L) was added into 1 mL of 40 µg/L ethyl acetate fraction and vortexed for 1 min. Prior to analysis, the samples were filtered through 0.45 μm membrane filters and the injection volume was 5 μL. The analysis was performed on a Thermo Scientific^TM^ Hypersil^TM^ BDS C18 Column (250 mm × 4.6 mm with 5 µm particles) at ambient temperature and the bioactive compounds of the extracts were detected at 280 nm. The mobile phases comprised a solution of deionization and distilled water + 0.5% acetic acid (Solvent A) and 100% acetonitrile (Solvent B). For separation, the following gradient elution system was used: 0–100 min, 95–70% A; 100–130 min, 70–95% A: 130–140 min, 95–95% A. In full-scan mode, the MS parameter was utilized with the following settings: spray voltage −4.5 kV, capillary temperature 270 °C, sheath gas flow rate 50 arb, auxiliary gas flow rate 20 arb, sweep gas flow rate 2 arb, and S-lens RF level 55.0. The LC–MS method was performed in full scan-data dependent Top5 (ddMS2/Top5) in negative mode (*m/z* 100–1200) with the following conditions: resolution 70,000; automatic gain control (AGC) target 3 × 10^6^; maximum ion injection times (max IT) 100 ms. The parameters of ddMS2 were as follows: resolution 17,500; AGC target 1 × 10^6^; max IT 50 ms; isolation window 2.0 amu, and normalized collision energy 33%.

Quantification was achieved based on the established calibration curve of luteolin (25–125 µg/L; y = 9336.5x + 13431; R^2^ = 0.9954). Results were represented as milligrams of luteolin equivalents per gram (mg/g).

### 2.12. Statistical Analysis

The experiment’s results were expressed as means ± standard deviations, and all tests were repeated thrice. The two-tailed Student *t*-test was used to determine the differences between two groups. To evaluate between-group differences, one-way ANOVA was used, followed by Duncan’s test using the SPSS statistical program (SPSS 24.0, SPSS Inc., Chicago, IL, USA). Differences were considered to be statistically significant when *p* < 0.05.

## 3. Results

### 3.1. Determination of TPC, TFC, and TAC

The phenolic compounds derived from natural products have various biological and pharmacological activities, as demonstrated in numerous studies [6,19]. The total phenolic contents of the black rice leaf extract and solvent fractions are described in Table 1. The TPC values of the CEE and various fractions were significantly different (*p* < 0.05), ranging from 106.83 to 303.54 mg GAE/g DW. Our results revealed that the TPC content in the CEE was higher than those of *n*-BuOH, EtOAc, aqueous, and Hex fractions. This was in agreement with recent research showing the content of TPC in the crude ethanolic extract was greater than the contents in the other solvent fractions for *Citrus hystrix* peel [20].

Flavonoids are a group of phenolic components found in plants that can reduce the risks of several chronic inflammatory diseases such as cancer, diabetes, and cardiovascular disease [21]. The TFC of various extracts was 54 to 405.23 mg QE/g DW; the extracts’ CEEs contained greater TFCs than their *n*-BuOH, EtOAc, aqueous, and Hex fractions, respectively. This suggested that a higher content of flavonoids is associated with a greater TPC content in the extract.

This study suggested that both ethyl acetate and *n*-butanol had intermediate polarity and thus greater efficacy in the extraction of moderately polar phenolics and flavonoids. The general concept of solubility, “like dissolves like”, can explain the fact that solvent extraction with solvents of different polarities can separate different types of phytochemical substances, such as phenolic compounds [22].

The anthocyanin values of the different extracts were between 52.33 and 344 mg C3GE/100g DW. Among the fractions obtained with solvents of different polarities, we observed that the *n*-BuOH fraction possessed the greatest TAC content, followed by the aqueous fraction. However, the Hex and EtOAc fractions exhibited low anthocyanin contents. This may be due to the high polarity of the anthocyanin molecules in black rice leaves, meaning they are better extracted with moderately to highly polar solvents such as *n*-butanol and aqueous solvents than with lower-polarity solvents (ethyl acetate and hexane). Moreover, the TAC trend was similar to the trends for the TPC and TFC, suggesting that anthocyanins belong to the phenolic hydroxyl group. A few recent studies found that pigmented rice cultivars had higher TPC, TFC, and TAC contents than non-pigmented cultivars; the differences in these values probably depend on the varieties of rice [13].

### 3.2. Antioxidant Activities of CEE and Its Fractions

The DPPH and ABTS free radical-scavenging activities and reducing powers of all the fractions and standard antioxidants (BHA and Trolox) are presented, as IC_50_ values (a smaller IC_50_ value corresponds to greater antioxidant capacity), in Table 2.

The CEE demonstrated the lowest IC_50_ value (12.32 µg/mL), followed by the *n*-BuOH (15.64 µg/mL), aqueous (32.38 µg/mL), EtOAc (32.79 µg/mL), and Hex (57.13 µg/mL) fractions. These data indicate that a stronger DPPH radical-scavenging activity was related with a higher TPC and TFC of the extract, as well as TAC. A previous study reported an excellent correlation between DPPH radical-scavenging capacity and phytochemical (i.e., phenolics and flavonoids, including anthocyanins) constituents [23]. Moreover, the presence of different antioxidant components in each fraction may explain the differences in the scavenging ability of the extracts against DPPH radicals [24].

For the investigation of antioxidant activity by the ABTS assay, we compared the antioxidant capacities of the extracts from various fractions with the synthetic antioxidant (Table 2). The results illustrate that the IC_50_ values of the CEE and other fractions were in the range of 3.22–55.04 μg/mL. These results showed a similar tendency for DPPH radical-scavenging ability among all the fractions; the *n*-BuOH and aqueous fractions revealed the strongest ABTS^+•^ scavenging capacities. These observations were in accordance with those for an *n*-BuOH *Ruta chalepensis* fraction, which demonstrated the highest antioxidant activity [25]. Previous reports have indicated that phenolics and anthocyanins are the predominant scavengers of the ABTS^+^ radical in black rice bran [17,26]. This may be due to not only the presence of higher TPC and TFC (especially chlorogenic acid), but also the anthocyanin contents in the *n*-BuOH and aqueous fractions, as phenolic, flavonoid, and anthocyanin compounds play essential roles as antioxidants in living organisms [27]. Thus, there is an excellent correlation between the antioxidant ability and the existence of these compounds. Numerous reports have shown that plants rich in phenolic components demonstrate strong antioxidant activities [6,25]. Although the EtOAc fraction exhibited high TPC and TFC values (similar to *n*-BuOH), it did not show the best antioxidant ability, which might be attributable to its relatively low TAC content.

As seen in Table 2, the *n*-BuOH fractions once again showed the greatest performance in the reducing power assay (405.27 µg/mL), while the lowest activity was found in the hexane fraction (1253.67 µg/mL). The different polarities of the solvents changed the extraction efficiency for specific antioxidant constituents, thus affecting the antioxidant properties of the extracts [22]. In this study, the sample extracts exhibited a homologous trend in both the radical-scavenging assays (DPPH and ABTS), with an enhancement in the reducing power observed as the IC_50_ values decreased.

### 3.3. Anti-Inflammatory Activities of CEE and Its Fractions in RAW 264.7 Macrophage Cells

**Figure 1:**
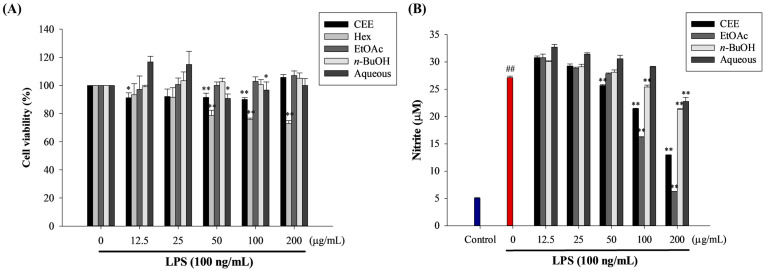
Effect of black rice leaf CEE and its solvent fractions on the viability of (**A**) and the production of nitric oxide (NO) by (**B**) RAW 264.7 cells stimulated with LPS. ^##^ *p* < 0.01, compared with non-stimulated LPS; * *p* < 0.05 and ** *p* < 0.01 compared with 100 ng/mL, LPS-stimulated.

The cytotoxic effects of the black rice leaf crude extract and its fractions were evaluated in RAW 264.7 cells using an MTT assay. The extract showed no cytotoxicity on macrophage cells at a high concentration, up to 200 µg/mL (Figure 1A). However, cell viability was reduced by 72% in macrophage cells by 50 µg/mL Hex (<80% of cell viability is considered as cytotoxicity [28]). Therefore, the CEE, EtOAc, *n*-BuOH, and aqueous fractions with concentrations between 12.5 and 200 µg/mL were selected for the following cell experiments.

Nitric oxide (NO) is an essential inflammatory mediator that induces inflammation. The results demonstrated that the best suppression effect on NO secretion induced by LPS was found by EtOAc and CEE, whereas the other fractions slightly down-regulated NO production (Figure 1B). Our results were correspondent with a previous report that revealed a group of flavone glycosides to be the major phenolic compounds found in rice leaf extract from Thailand. It attributed to the blocking of LPS-activated NO secretion in macrophage cells, RAW 264.7, in a concentration-dependent manner (100–2000 µg/mL); the inhibition ranged from 40 to 85% [29]. Moreover, flavonoid compounds, luteolin, orientin, rutin, and kaempferol also have been shown to suppress the production of NO in response to inflammatory stimuli by inhibiting iNOS expression [30,31,32]. Considering these in vitro results, the CEE and EtOAc fraction were selected for further anti-inflammatory experiments.

### 3.4. Effect of CEE and EtOAc on LPS-Induced PGE_2_ and Cytokine Production in RAW 264.7 Macrophage Cells

**Figure 2:**
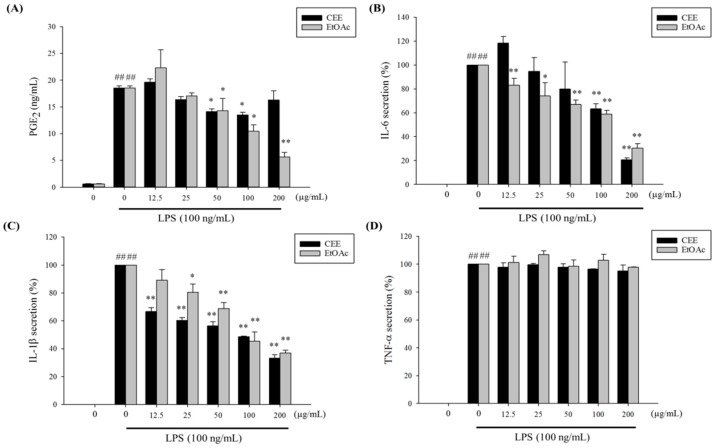
Effects of CEE and EtOAc fractions on PGE_2_ (**A**), IL-6 (**B**), IL-1β (**C**), and TNF-α (**D**) production in LPS-induced RAW 264.7 cells. ^##^ *p* < 0.01, compared with non-stimulated LPS; * *p* < 0.05 and ** *p* < 0.01 compared with 100 ng/mL, LPS-stimulated.

After the pre-treatment of macrophage cells with 100 ng/mL LPS for 24 h, the production of PGE_2_ was significantly higher (*p* < 0.05) than that without LPS stimulation (Figure 2A). We observed that CEE and EtOAc effectively inhibited the PGE_2_ secretion induced by LPS in a dose-dependent manner. The EtOAc fraction was able to suppress PGE_2_ production better than CEE, which was consistent with the fact that a wide variety of phenolic substances were found in the EtOAc fraction as showed in Table 3. Thus, it might be associated with their anti-inflammatory activity in RAW 264.7 cells. In agreement with earlier studies, polyphenols can inhibit the secretion of PGE_2_ by the downregulation of the expression of COX-2 protein in murine and mice macrophages [33,34].

Cytokines such as IL-6, IL-1β, and TNF-α play essential roles in the inflammatory and immune responses. The numerous biological functions of IL-6 include controlling the immune system, regenerative processes, metabolism, and bone homeostasis; protecting the cardiovascular system; and preserving the functioning of the nervous system [35]. IL-1β is a crucial inflammatory-response mediator that is central to host responses to infection and injury [36]. TNF-α is regarded as a critical pro-inflammatory cytokine and immunomodulatory molecule that occurs in the acute phases of inflammation and infection [37]. As shown in Figure 2B,C, the ELISA results illustrated that the CEE and EtOAc (12.5–200 µg/mL) significantly (*p* < 0.05) deducted the secretion of pro-inflammatory cytokine (IL-6 and IL-1β) triggered by LPS, in a concentration-dependent manner. The suppression of IL-6 and IL-1β secretion by CEE at high concentrations was greater than that of EtOAc. Our recent studies showed that the CEE fraction contains high phytochemical contents and has an antioxidant capacity greater than that of the EtOAc fraction. Previous studies have demonstrated that phenolic-rich extracts from medicinal herbs and dietary plants, including fruits and vegetables, reduce the release of IL-6 and IL-1β, thus enabling the development and activation of the innate and adaptive immune system [33,38]. Furthermore, IL-6 and IL-1β are both involved in a broad range of other biological activities, regenerative processes, metabolic control, and the prevention of cardiovascular disease [35,36]. On the other hand, a slight downregulation of TNF-α secretion by LPS-induced RAW 264.7 cells was only observed under a high dose of the CEE (Figure 2D). A previous study showed similar results with lycopene, demonstrating powerful anti-inflammatory activities in activated macrophages [39]. Lycopene blocked the expression of IL-1β and IL-6 induced by LPS, mainly affected by ERK1/2, but was unable to suppress TNF-α (which is essentially controlled by JNK). Phenolic-rich mushroom extracts have also been shown to inhibit the secretion of NO, IL-1β, and IL-6 but have little effect on TNF-α release [40]. Our results were similar to a previous finding that phenolic-rich extracts from common beans reduced the levels of IL-1β and IL-6 but were unable to suppress TNF-α mRNA expression in LPS-activated RAW 246.7 cells [41]. Thus, these results indicate that the ant-inflammatory effect observed could be due to the selective suppression of different inflammatory factors in LPS-stimulated macrophages.

### 3.5. Effect of CEE and EtOAc on LPS-Induced iNOS and COX-2 Expression in RAW 264.7 Macrophage Cells

**Figure 3:**
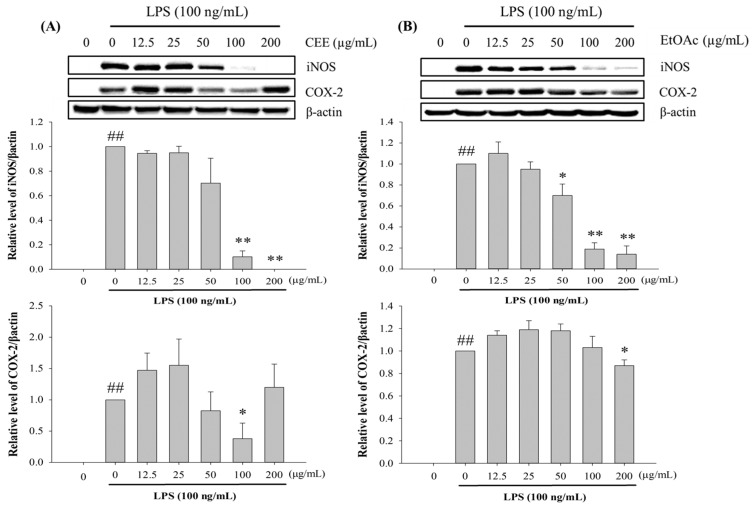
Effects of CEE (**A**) and EtOAc (**B**) fractions on iNOS and COX-2 protein secretion in LPS-induced RAW 264.7 cells. ^##^ *p* < 0.01, compared with non-stimulated LPS; * *p* < 0.05 and ** *p* < 0.01 compared with 100 ng/mL, LPS-stimulated.

To evaluate whether the phenolic-rich CEE and EtOAc fractions suppressed LPS-induced iNOS and COX-2 production in RAW 264.7 cells, the cells were pre-treated with or without the CEE and EtOAc fractions (12.5–200 µg/mL) for 4 h, and then activated with LPS for 20 h (Figure 3). The expression of the iNOS and COX-2 proteins, assessed by Western immunoblot assays, was enhanced by LPS stimulation. Nevertheless, the pre-treatment of the macrophage cells with the CEE or EtOAc fraction decreased the iNOS enzyme activity in a concentration-dependent manner (Figure 3A). We also observed that the CEE inhibited iNOS expression better than the EtOAc fraction. iNOS expression was dramatically reduced by the CEE at 100 and 200 µg/mL, with inhibition rates of 89.83% and 100%, respectively. This suggests that the reduction of NO secretion by the CEE and EtOAc fractions was a consequence of suppressing iNOS, a crucial enzyme for generating nitric oxide under physiological and pathological conditions [42]. In addition, the EtOAc fraction was able to inhibit COX-2 protein expression in LPS-activated macrophage cells only at 200 µg/mL (13.17%; Figure 3B). These results display that the suppression of PGE_2_ levels was probably due to the downregulation of COX-2 protein secretion [34]. These observations indicate that the ability to inhibit iNOS and COX-2 protein expression might be related to the anti-inflammatory properties of black rice leaves. Bioactive components, such as phenolics and anthocyanins, have been reported to possibly play a critical function in the anti-inflammatory effect, through reducing the expression of these two proteins [6]. A previous study revealed that luteolin and luteoloside, the main active components of ethyl acetate fraction derived from *Taraxacum coreanum*, could block the LPS/IFN-γ-induced secretion of NO, NF-κB activation, as well as iNOS and COX-2 expression [43]. Cyanidin-3-O-β-d-glycoside, cyanidin, and protocatechuic acid, which are major components of black rice extracts, were found to be potent inhibitors of not only pro-inflammatory cytokines but also iNOS and COX-2 expression in LPS-stimulated RAW 264.7 cells and carrageenan-triggered inflammation in BALB/c mice [44].

### 3.6. Anticancer Activities of CEE and Its Fractions on HepG2 Cells

**Figure 4:**
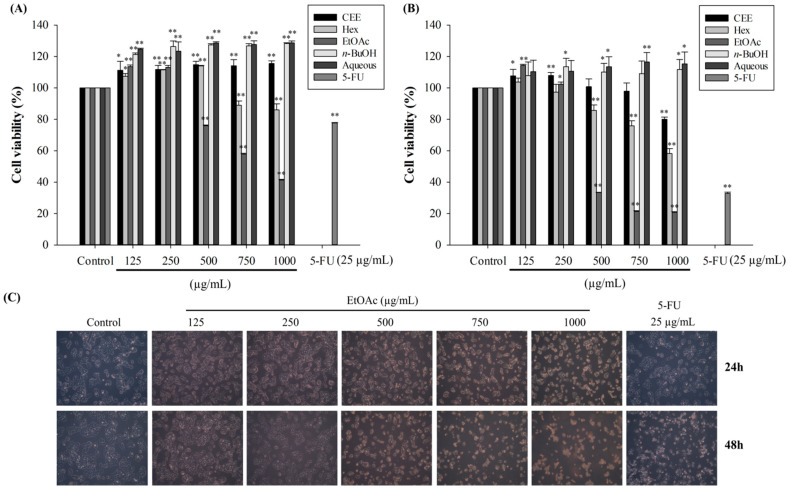
Antiproliferative effects of CEE and various solvent fractions on HepG2 cells. Cell growth was determined after treatment with 0–1000 µg/mL of CEE and solvent fractions for 24 h (**A**) and 48 h (**B**) using the MTT assay. (**C**) Cell morphology of HepG2 cells was observed by inverted microscopy after treatment with EtOAc fraction for 24 and 48 h. * *p* < 0.05 and ** *p* < 0.01, significant difference between the control and the concentration of each sample.

We measured the effects of the black rice leaf crude extract and its fractions on the viability of the HepG2 hepatocellular carcinoma and FL83B normal liver cell lines. All the extracts were non-toxic to normal FL83B cells after incubation for 48 h (Figure S1). As shown in Figure 4A,B, the ethyl acetate fraction suppressed the proliferation of HepG2 cells the most after 24 and 48 h. The Hex and EtOAc fractions inhibited the growing of cells in a dose- and time-dependent manner, although the HepG2 cell viability was unaffected by the *n*-BuOH or aqueous fractions. In addition, the EtOAc fraction, at multiple concentrations (750 and 1000 µg/mL), was much more inhibitory than 25 µg/mL 5-fluorouracil (5-FU), a clinical medicine that was regarded as a positive control for HepG2 cell treatment. The greater antiproliferative activities of the ethyl acetate fraction against the HepG2 cells may be explained by its higher number of active compounds than the other fractions. Many previous studies have demonstrated that polyphenol components extracted with ethyl acetate were observed in greater proportions than hexane, chloroform, and *n*-butanol [20,45] and can exert strong chemoprotective properties through suppressing the growth of various cancer cells, such as colon, breast, and liver cancer cells, by triggering both intrinsic and extrinsic apoptosis pathways [46]. As presented in Figure 4C, the morphology of the cells changed after incubation with different concentrations of the EtOAc fraction at 24 and 48 h. These cellular changes, such as cell body shrinkage, reduced cell adherence, decreased cell populations, and increased rates of cell death, are characteristic of the induction of apoptosis [47], indicating that apoptosis could be the main factor contributing to the suppression of cancer cell growth. Based on the above results, treatments with 250, 500, 750, and 1000 µg/mL of the EtOAc fraction were selected for further experiments.

**Figure 5:**
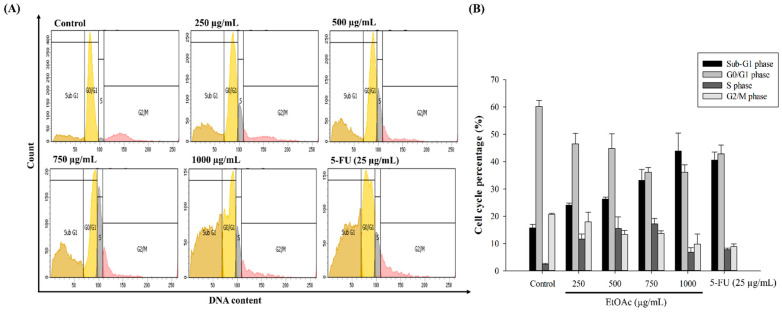
The effect of the EtOAc fraction on Sub-G1 phase accumulation and cell-cycle arrest in HepG2 cells. The cells were treated with 0–1000 µg/mL of the EtOAc fraction for 48 h and stained with PI, followed by flow cytometry (**A**). Data represent the percentages of cell proportion in each cell-cycle phase (Sub-G1, G0/G1, S, and G2/M) (**B**).

To further investigate the effects of the inhibition of HepG2 cell growth and proliferation, we measured the regulation of cell-cycle progression using flow cytometry. The cell cycle was categorized, by cell population, into four different phases: Sub-G1, G0/G1, S, and G2/M. The accumulation of a Sub-G1 population is considered a feature of apoptotic cell death. According to Figure 5, the population of cells in the Sub-G1 phase significantly increased after the HepG2 cells were treated with 250 (24.03%), 500 (26.23%), 750 (33.13%), and 1000 (43.90%) µg/mL of the EtOAc fraction or 25 µg/mL 5-FU (40.57%) for 48 h, compared with the control (15.70%). Moreover, the EtOAc fraction induced cell-cycle arrest at the S phase and decreased cell accumulation in the G0/G1 phase. This finding suggests that the EtOAc fraction exerted its suppressive activity through the inhibition of cell proliferation by triggering apoptosis and Sub-G1 accumulation in a concentration-dependent manner. According to previous studies, dietary bioactive constitutes, such as phenolic acids and flavonoids can downregulate cell proliferation by the obstruction of cell-cycle progression at the G0/G1, S, or G2/M phase, as well as increasing the Sub-G1 population [46,48].

**Figure 6:**
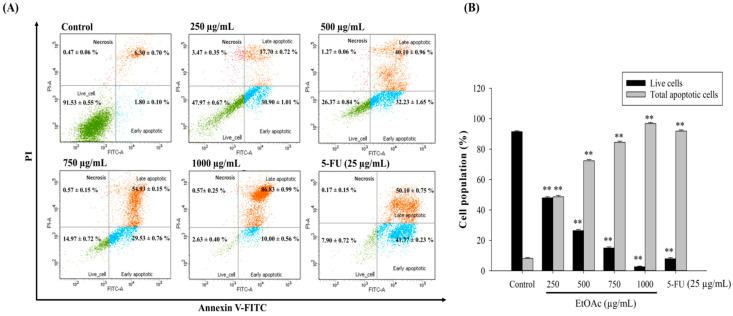
The effect of the EtOAc fraction on HepG2 cell apoptosis. The cells were treated with 0–1000 µg/mL of the EtOAc 48 h and labeled with Annexin V-FITC and PI, followed by the measurement of cell apoptosis by flow cytometry (**A**). The bar chart presents the percentage of live cells and total apoptotic cell population of HepG2 cells (**B**). ** *p* < 0.01, significant difference between the control and the concentration of the EtOAc fraction.

To confirm whether the EtOAc fraction triggered cell apoptosis, we measured the apoptotic cell death of HepG2 cells treated with 250–1000 µg/mL of the ethyl acetate fraction or 5-FU (25 µg/mL) for 48 h, using Annexin-V/PI labeling in flow cytometry. As presented in Figure 6, the EtOAc fraction reduced the percentage of live cells and enhanced that of apoptotic cells in a dose-dependent manner. The proportion of total cell apoptosis (the sum of early and late apoptosis) significantly increased from 8.10% ± 0.61% in the control group to 48.60 ± 0.95% (250 µg/mL), 72.33 ± 0.84% (500 µg/mL), 84.47 ± 0.85% (750 µg/mL), and 96.83 ± 0.71% (1000 µg/mL), while the standard (5-FU, 25 µg/mL) resulted in a 91.87 ± 0.81% apoptotic population (Figure 6B). We also found that the EtOAc fraction induced cell death more through late-phase than early-phase apoptosis (Figure 6A). In a recent study, an enhancement of apoptosis at the late phase was observed for lettuce-extract-treated Caco-2 cells [49]. Thus, the data indicate that the ethyl acetate fraction could trigger apoptosis in liver cancer cells.

### 3.7. Key Proteins in Apoptotic Pathways Regulated by EtOAc Fraction

**Figure 7:**
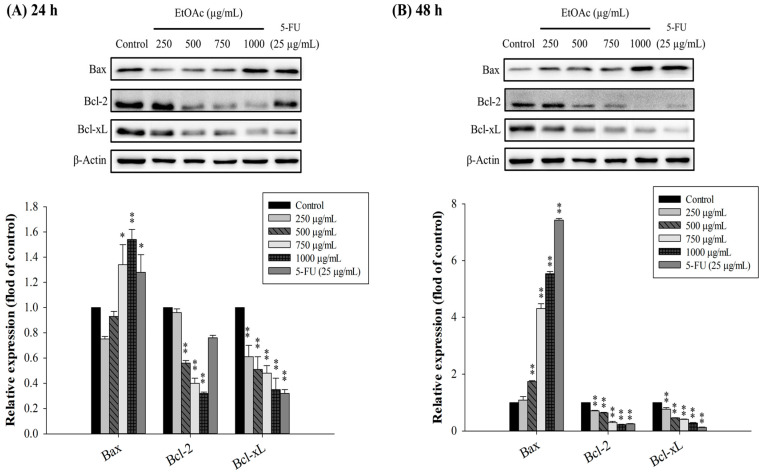
Effect of EtOAc fraction on the expression of Bcl-2 family proteins in HepG2 cells. The cells were treated with 0–1000 µg/mL of the EtOAc fraction for 24 h (**A**) and 48 h (**B**). * *p* < 0.05 and ** *p* < 0.01, significant difference between the control and the concentration of the EtOAc fraction. β-actin was used in Western blot analysis as a loading control.

**Figure 8:**
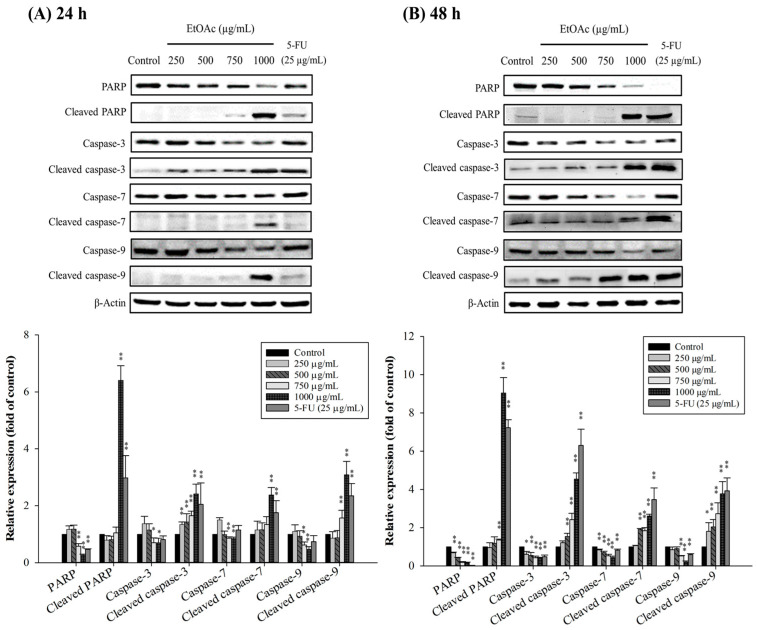
Effect of EtOAc fraction on the expression of apoptotic proteins (PARP, cleaved PARP, and caspase family) in HepG2 cells. The cells were treated with 0–1000 µg/mL of the EtOAc fraction for 24 h (**A**) and 48 h (**B**). * *p* < 0.05 and ** *p* < 0.01, significant difference between the control and the concentration of the EtOAc fraction. β-actin was used in Western blot analysis as a loading control.

In apoptosis, a variety of related proteins are involved. We thus assessed the apoptosis-related proteins by Western blotting to prove that the ethyl acetate fraction triggered apoptosis in HepG2 cells. Bcl-2 family proteins have a significant influence on the regulation of the mitochondrial apoptosis pathway, by controlling the activation of caspases [50]. Caspases are mainly involved in mediating apoptotic-signal transduction and can be categorized, by their mechanism of action, into initiator (caspase-2, -8, -10, and -9) and executioner caspases (caspase-3, -6, and -7) [51]. The Western blotting revealed that the EtOAc-treated HepG2 cells showed an increase of a pro-apoptotic protein, Bax, and reduction of anti-apoptotic proteins, Bcl-2 and Bcl-xL, in a dose- and time-dependent manner following incubation times of 24 and 48 h, compared with untreated control cells (Figure 7A,B). As the concentration of the EtOAc fraction was increased, caspase-3, -7, and -9 expression decreased and the activities of cleaved caspase-3, -7, and -9 increased (Figure 8A,B). Poly(ADP-ribose) polymerase (PARP) is a particular substrate for the executioner caspase-3 and caspase-7, and is cleaved into its active form during apoptosis [52]. Figure 8A,B also demonstrate that the EtOAc fraction enhanced the activation of PARP cleavage in a dose- and time-dependent manner, indicating that the caspase-mediated pathway was related to EtOAc-induced apoptosis in HepG2 cells. Our findings were correspondent with previous reports demonstrating that natural phenolics, such as protocatechuic acid, luteolin, rhamnazin, and orientin, can cause apoptosis in various cancer cells, including hepatocarcinoma cells, by the mitochondrial apoptosis pathway [46,48].

### 3.8. Composition of Bioactive Compounds

**Figure 9:**
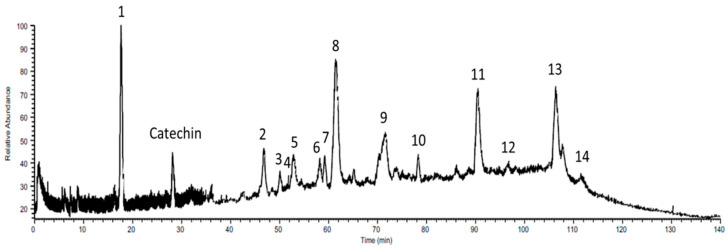
Total ion chromatogram of the EtOAc fraction–Catechin 20 ppm produced by UV-LC-MS/MS with UV detection at 280 nm and electrospray ionization negative full scan mode. The peak numbers in the figure correlated to those in Table 3 below.

The polyphenol profiles of ethyl acetate fraction derived from black rice leaf displaying outstanding anti-inflammatory and anticancer capacities were evaluated and quantified for the first-time using UV-LC-MS/MS analysis. Total ion chromatogram with UV detection at 280 nm and the contents of bioactive compounds of this fraction were presented in Figure 9.

Numerous flavonoid components with essential biological activities were detected in the ethyl acetate fraction (Table 3). Orientin, 22.89 mg/g (peak 2), 3,3,4,5,5,7 hexahydroxyflavanone, 8.51 mg/g (peak 3), isoquercitrin, 11.58 mg/g (peak 6), luteolin 7-O-glucoside, 16.26 mg/g (peak 7), luteolin, 21.79 mg/g (peak 11), and rhamnazin, 25.63 mg/g (peak 13), were identified in EtOAc fraction. Orientin, also called luteolin-8-C-glucoside, is found in various medicinal plants; it possesses many bioactive effects, antioxidant, antiaging, antibacterial, antiviral, anti-inflammatory, radioprotective, and antitumor effects [53]. Isoquercitrin (quercetin-3-glucoside) is a phytochemical found in several plants, which exhibits an outstanding capacity for alleviating allergic reactions, viruses, inflammation, cancer progression, and free radical damage [54]. Luteolin and luteolin 7-O-glucoside suppressed NO and PGE_2_ production and also downregulated the secretion of iNOS and COX-2 in LPS-activated RAW 264.7 cells via NF-κB/AP-1/PI3K-Akt signaling pathway [55]. Moreover, these compounds exhibited antibacterial, antifungal, antioxidant properties, and the ability to suppress proliferation, migration, and angiogenesis in several forms of cancer cell lines such as lung, brain, breast, colorectal, prostate, and pancreatic. [56,57]. Studies have shown that rhamnazin (O-methylated flavonol) is abundant in *Rhamnus petiolaris*, *Ginkgo biloba*, *Salix*, and other medicinal herbs, which plays a critical role in pharmaceutics; it also has antioxidant, antimutagen, anti-inflammatory, and anticancer properties, as well as other biological activities [58].

Furthermore, protocatechuic acid, 18.51 mg/g (peak 1), sinapic acid, 8.81 mg/g (peak 4), and hydroxygallic acid, 7.65 mg/g (peak 9), were identified as the major phenolic acids in the EtOAc fraction. A previous study reported that protocatechuic acid, a natural phenolic acid found in numerous diets, including vegetables, fruits, teas, and grains, significantly decreased the expression and production of inflammatory mediators in RAW 264.7 and BV-2 cells stimulated with LPS through NF-κB and MAPKs pathways, and can increase the suppression of tumor cell growth and induce caspase-mediated apoptosis on many types of cancer cells [59]. Sinapic acid is one of the most hydroxycinnamic acids in a broad range of plants with medicinal benefits in chronic inflammatory disease and anti-oxidant, anti-inflammatory, and anti-cancer properties [60]. However, the bioactivity of hydroxygallic acid as components in pomegranate and vine shoot has not been reported [61,62].

Many studies have demonstrated that ethyl acetate, as one of the moderately polar solvents, tends to be more efficient than others in the extraction of polyphenols [20]. Our findings corroborate a former study that discovered a high concentration of phenolic substances in the ethyl acetate fraction of purple perilla leaf [45].

**Table 3:** Phenolic compounds identified in EtOAc fraction by UV-LC-MS/MS.

Peak	Retention Time (min)	[M − H]^−^ (*m/z*)	MS/MS Production (Relative Abundance)	Compounds	Amount (mg/g) ^1^	References
1	17.68	153	109(100.0%), 153(39.6%)	Protocatechuic acid	18.51 ± 0.35	[63]
2	46.75	447	297(14.7%), 327(100.0%), 357(90.7%)	Orientin	22.89 ± 1.33	[64]
3	50.16	319	95(7.0%), 139(41.9%), 153(21.1%), 183(100.0%)	3,3,4,5,5,7 Hexahydroxyflavanone	8.51 ± 0.38	[65]
4	52.94	223	164(53.8%), 208(100.0%), 223(20.6%)	(E)-Sinapic acid	8.81 ± 1.82	[63]
5	54.51	755	163(28.3%), 205(29.8%), 309(100.0%), 357(34.4%), 429(53.7%), 489(11.5%)	Unknown	8.66 ± 0.21	-
6	58.48	463	257(19.6%), 300(100.0%), 463(3.6%)	Isoquercitrin	11.58 ± 0.41	[63]
7	59.23	447	285(100.0%), 447(15.5%)	Luteolin 7-O-glucoside	16.26 ± 0.40	[63]
8	65.20	369	145(100.0%), 163(39.5%), 205(15.6%), 309(38.0%), 351(5.3%)	Unknown	17.01 ± 0.14	-
9	71.39	187	125(100.0%), 187(51.8%)	Hydroxygallic acid	7.65 ± 0.79	[61]
10	78.37	795	256(18.2%), 271(100.0%)	Unknown	16.09 ± 0.43	-
11	90.46	285	65(9.4%), 107(19.3%), 133(100.0%), 151(38.7%), 175(20.1%), 199(15.5%), 217(9.1%), 285(49.2%)	Luteolin	21.79 ± 0.45	Standard
12	96.58	747	313(24.3%), 328(100.0%)	Unknown	9.03 ± 0.95	-
13	106.45	329	299(51.3%), 314(100%), 329(46.9%)	Rhamnazin	25.63 ± 0.82	[66]
14	111.68	585	165(79.4%), 195(16.9%), 314(53.1%), 329(100.0%)	Unknown	21.64 ± 2.91	-

^1^ Amounts of compounds 1 to 14 were presented as milligrams of luteolin equivalents per gram.

## 4. Conclusions

Agricultural residues have become an increasingly concerning issue worldwide in recent years, as they can potentially lead to increased environmental pollution. In this study, black rice leaf extracts were shown to contain high phytochemical contents, corresponding to enhanced antioxidant capacities. Remarkably, the anti-inflammatory and anticancer activities of black rice leaves were reported for the first time. The CEE and its fractions also inhibited the production of NO. The CEE and EtOAc fractions suppressed PGE_2_, IL-6, and IL-1β secretion, as well as decreasing the expression of iNOS and COX-2. Moreover, the EtOAc fraction was shown to inhibit HepG2 cancer cell growth by triggering apoptosis through the mitochondrial pathway. These findings indicated that black rice leaves should be considered as a potential source of natural antioxidant, anti-inflammatory, and anticancer agents for nutraceutical and pharmaceutical applications. Future studies are required, such as animal and clinical studies.

## Figures and Tables

**Table 1:** TPC, TFC, and TAC contents of black rice leaf extract and fractions.

Fractions	TPC (mg GAE/g DW)	TFC (mg QE/g DW)	TAC (mg C3GE/100g DW)
Crude ethanolic extract	303.54 ± 2.06 ^a^	405.23 ± 2.26 ^a^	344.00 ± 5.79 ^a^
Hexane	106.83 ± 0.84 ^e^	54.00 ± 2.00 ^d^	52.33 ± 2.55 ^d^
Ethyl acetate	226.83 ± 0.84 ^c^	220.00 ± 2.00 ^b^	48.43 ± 1.67 ^d^
Normal butanol	233.78 ± 1.92 ^b^	223.33 ± 2.31 ^b^	212.08 ± 1.67 ^b^
Aqueous	137.11 ± 1.27 ^d^	137.33 ± 2.31 ^c^	112.44 ± 1.93 ^c^

TPC: total phenolic content; TFC: total flavonoid content; TAC: total anthocyanin content; GAE: garlic acid equivalent; QE: quercetin acid equivalent; C3GE: cyanidin-3-glucoside equivalent. The phytochemical contents are shown as mean ± SD (*n* = 3). Means within the same column with various superscripts illustrate significant differences (*p* < 0.05).

**Table 2:** IC_50_ values of antioxidant activities of black rice leaf crude extract and fractions.

Fractions	DPPH^•^ (µg/L)	ABTS^•+^ (µg/L)	Reducing Power (µg/L)
Crude ethanolic extract	9.77 ± 0.06 ^e^	175.30 ± 0.69 ^d^	324.74 ± 1.51 ^e^
Hexane	57.13 ± 0.24 ^a^	627.74 ± 1.68 ^a^	1253.67 ± 3.76 ^a^
Ethyl acetate	32.79 ± 0.09 ^b^	273.65 ± 1.05 ^b^	682.06 ± 2.02 ^b^
Normal butanol	15.64 ± 0.09 ^d^	212.75 ± 0.78 ^c^	332.00 ± 1.28 ^d^
Aqueous	32.28 ± 0.23 ^c^	213.16 ± 0.37 ^c^	405.27 ± 0.76 ^c^
BHA	4.46 ± 0.06 ^f^	nd	nd
Trolox	nd	52.04 ± 0.10 ^e^	nd
Ascorbic acid	nd	nd	49.63 ± 0.66 ^f^

BHA: butylated hydroxyanisole; trolox: 6-hydroxy-2,5,7,8-tetramethylchroman-2-carboxylic acid. Values are displayed as mean ± SD (*n* = 3). Means within the same column with various superscripts illustrate significant differences (*p* < 0.05). nd: not detected.

## Data Availability

Data is contained within the published article.

## Supplementary Materials

The following are available online at https://www.mdpi.com/article/10.3390/foods10122987/s1, Figure S1: Cell viability of CEE and solvent fractions on FL83B cells were measured using MTT assay. Cells were treated various concentrations (0–1000 μg/mL) of CEE and sovent fractions for 48 h.
