# Supplementary material for: Evaluation of Phytochemical Contents and In Vitro Antioxidant, Anti-Inflammatory, and Anticancer Activities of Black Rice Leaf (Oryza sativa L.) Extract and Its Fractions
Source: Foods. 2021 Dec 3;10(12):2987. doi: 10.3390/foods10122987 (PMC8701243; doi:10.3390/foods10122987)
Supplement: Supplementary file 1 [file foods-10-02987-s001.zip › foods-1441342-supplementary.pdf]

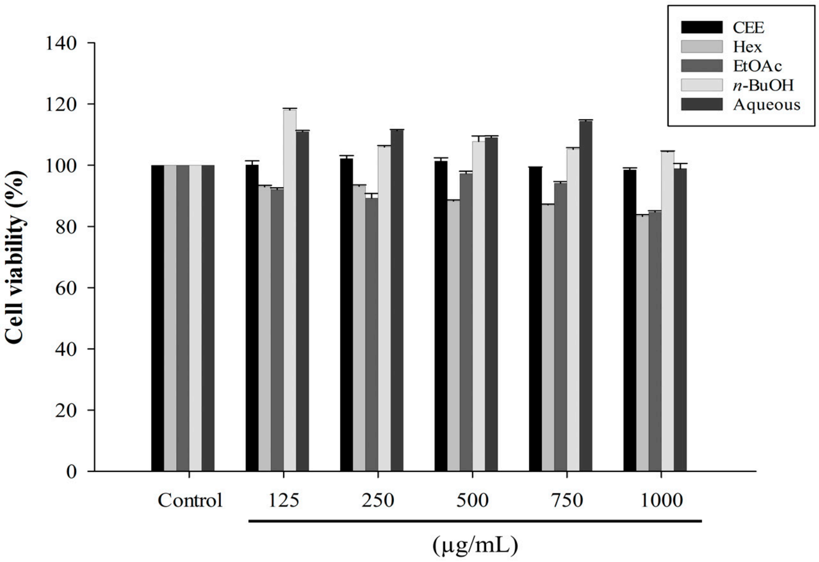

Figure S1. Cell viability of CEE and solvent fractions on FL83B cells were measured using MTT assay. Cells were treated various concentrations (0 – 1000 μg/mL) of CEE and sovent fractions for 48h.
